# Fabrication of Functional Carbon/Magnetic Nanocomposites as A Promising Model of Utilization of Used Crosslinked Polymers

**DOI:** 10.3390/ma11122595

**Published:** 2018-12-19

**Authors:** Rasim Alosmanov, Jennet Imanova, Karol Wolski, Ralf Ziemmermann, Sylwia Fiejdasz, Janusz Przewoźnik, Kamil Goc, Czesław Kapusta, Szczepan Zapotoczny, Michał Szuwarzyński

**Affiliations:** 1 Chemistry Department, Baku State University, Z. Khalilov Str. 23, AZ1148 Baku, Azerbaijan; r_alosmanov@rambler.ru (R.A.), kerimovacennet@gmail.com (J.I.); 2 Faculty of Chemistry, Jagiellonian University, Gronostajowa 2, 30-387 Krakow, Poland; wolski@chemia.uj.edu.pl (K.W.), zapotocz@chemia.uj.edu.pl (S.Z.); 3 Joint Mass Spectrometry Centre, Helmholtz Zentrum Munchen, German Research Center for Environmental Health (GmbH), Ingolstadter Landstrasse 1. D-85764 Nauherberg, Germany; ralf.zimmermann@helmholtz-muenchen.de; 4 Joint Mass Spectrometry Centre, Institute of Chemistry, University of Rostock, Dr. Lorenz Weg 1. D-18051 Rostock, Germany; 5 Faculty of Physics and Applied Computer Science, Department of Solid State Physics, AGH University of Science and Technology, Mickiewicza 30, 30-059 Krakow, Poland; fiejdasz@agh.edu.pl (S.F.), januszp@agh.edu.pl (J.P.), kamil.goc@fis.agh.edu.pl (K.G.), kapusta@agh.edu.pl (C.K.); 6 Academic Centre for Materials and Nanotechnology, AGH University of Science and Technology, Mickiewicza 30, 30-059 Krakow, Poland

**Keywords:** phosphorus-containing polymer, thermal degradation, carbon/magnetic composite, nanoparticles

## Abstract

The utilization of used crosslinked functional polymers (CFP) applied as sorbents or ion-exchangers is a great challenge arising from the need to protect the environment. In this paper we report a very promising way of obtaining carbon/magnetic composites based on metal (Co^2+^; Ni^2+^; Fe^3+^) derivatives of butadiene rubber-based phosphorus-containing polymer, which were treated as the model used CFP. We proposed a facile one-step thermal degradation approach to transform used CFP into carbon/magnetic composites (CMC). The obtained CMCs contained a mixture of metal phosphates and metal phosphides that exhibited strong magnetic properties due to the presence of nanosized metal derivatives with diameters of 100–140 nm. Structural and morphological changes of CFP and CMC after thermal degradation were investigated by the FTIR technique, X-ray Diffraction analysis, Scanning Electron Microscope, and Atomic Force Microscope–Magnetic Force Microscope. Moreover, thermal degradation kinetics parameters were determined to optimize the efficiency of the process.

## 1. Introduction

Crosslinked functional polymers (CFP) as sorbents and ion-exchange resins have been widely used in the production of demineralized and deionized water [1], boiler feed water in high pressure steam generators [2], in the treatment of waste water originating from the plating industry or nuclear power stations [3,4,5], and as solid acid catalysts applied for metal separation from aqueous solutions [6]. The reuse of used CFP with adsorbed metal ions requires regeneration of the polymeric material by the desorption process of metal ions using various acid [7,8], base or salt solutions [9]. However, such treatments have serious disadvantages. First of all, the number of sorption and desorption cycles is limited due to deterioration of the chemical and physical properties of the CFP after each cycle [10]. Secondly, the solutions used for the regeneration become contaminated with metal ions and also need to be disposed. Thus, in some cases the costs of regeneration process are too high and economically unprofitable in comparison to purchasing new, non-used polymers. Finally, some of the polymers cannot be used even after regenerations if e.g., they were loaded with radioactive nuclides [11]. Thus, the used CFP have been accumulating as solid wastes and are considered to be useless materials. Used CFP are mostly deposited in landfill sites where they may undergo degradation and release as hazardous species that could contaminate the environment [12]. Several approaches were proposed and investigated for utilization of used CFP such as: Burning with copper oxide catalyst [13], pyrolysis for reducing the waste volume and obtaining more stable final form of waste [14,15], application as a replacement of fuel in sintering processes [16], or usage as additives to coal for coke production [17]. Apparently, most of these methods lead to degradation of CFP while it would be more beneficial if some functional materials could be obtained instead. Moreover, the amount of used CFP residual after thermal degradation (TD) drops to the level of about 30–40% of the initial mass [18,19,20,21]. In this regard, a feasible approach for the utilization of used CFP may involve the preparation of various carbon materials (CM) such as activated carbon (AC). Various ACs have been obtained and used as: Adsorbents for various types of commercial resins [22], Amberjet 1200 H resins [23,24], and polysulfonated cation exchange resins [25,26]. Recent research has focused on the fabrication of AC/iron composites based on spent polystyrene resin [27]. Due to specifically designed multi-stage conditions (carbonization and activation in a horizontal cylindrical furnace under N_2_ flow) such composites contained zero-valent iron and iron oxides (Fe_3_O_4_, γ-Fe_2_O_3_), which ensured their magnetic properties. Nowadays, such materials, i.e., magnetic composites (MC), are considered as promising functional materials that have gained significant attention due to their inherent magnetic properties [28,29]. It is well known that the properties of CM are determined both by their structure and the chemical nature of their surface [30]. The spatial arrangement of the carbon atoms determines the texture and structure of CM, while the presence of various heteroatoms such as oxygen, nitrogen, or phosphorus, originating from the precursor or activation agent, changes their chemical properties [31,32]. CM with incorporated heteroatoms that ensure the magnetic properties keep the simplicity of their preparation using the thermal degradation methods and enable, e.g., their easy removal of the system just by applying a magnetic field.

In this paper a very promising, environmentally-friendly and resource-saving method of used CFP treatment leading to fabrication of carbon magnetic composites (CMC) was proposed. Metal derivatives of butadiene rubber-based phosphorus-containing polymer (Fe-PhCP, Co-PhCP, Ni-PhCP) were investigated as the model used CFP. Generally accepted, conventional TD without any modification was used as an approach for obtaining CMC. The studies of thermal behavior of the Fe-PhCP, Co-PhCP, Ni-PhCP, and kinetic analysis of the TD in comparison to initial PhCP materials are described in this work. Kinetic analysis allowed to reveal the optimum parameters of synthesis of CMCs. In addition, investigation of TD process in details disclosed the influence of metal ions on the TD process of PhCP. The organic nature and crosslinked structure of the polymer, the presence of phosphorus-containing functional groups and metal ions in the polymer structure enabled obtaining functional CMC. A structural and morphological characterization of CMC using SEM, XRD, and AFM-MFM was also performed and their magnetic character was confirmed.

## 2. Materials and Methods

### 2.1. Materials

Phosphorus-containing polymer (PhCP) in the hydrogen form as a starting material was synthesized via oxidative chlorophosphorylation reaction of butadiene rubber using the method described before [33]. The corresponding metals salts (Co(NO_3_)_2_⋅6H_2_O; Ni(NO_3_)_2_⋅6H_2_O; and FeCl_3_⋅6H_2_O) were used for preparing metal forms of PhCP. All salts were chemically pure (p.a.) and purchased from Gorex Analyt GmbH.

### 2.2. Preparation of PhCP Metal Forms

The metal forms of the PhCP were prepared by mixing of PhCP (0.3 g) with 90 ml of water solutions of corresponding metal salts (10 mM). Mixtures were left for shaking in a temperature-controlled shaker for 12 h at room temperature. Afterwards, the dark brown powder was filtered and dried in the air at room temperature (RT). The metal concentration in solutions before and after adsorption was controlled by means of atomic emission spectroscopy (Optima 2100 DV, Perkin Elmer, Waltham, MA, USA).

### 2.3. Fourier Transform Infrared (FTIR) Spectroscopy Analysis

The PhCP and all metal derivatives were characterized by FTIR using a Nicolet iS10 FTIR spectrometer (Thermo Fisher Scientific, Waltham, MA, USA) with ATR accessory (SMART iTX, Thermo Fisher Scientific). All samples were analyzed after vacuum drying. The obtained spectra were baseline corrected and normalized using Omnic v9.0 software (Thermo Fisher Scientific).

### 2.4. Thermal Degradation (TD)—Preparation of Carbon/Magnetic Composites

TD was performed on NETZSCH TG 209/cell Thermal Analysis System (Netzsch, Selb, Germany) working in the temperature range between 20 and 850 °C using Al_2_O_3_ crucibles. TD process for the carbon materials (CM) and carbon/magnetic composites (CMC) for granulated samples (the mass of each sample was equal to 10.16 ± 0.27 mg) was carried out in a synthetic air flow (35 mL·min^−1^) with heating rate of 1 K·min^−1^. Moreover kinetic analysis was performed at four different heating rates (β = 5, 10, 15, and 20 K·min^−1^). The results of TD processes were shown as thermo-gravimetric/differential gravimetric (TG/DTG) curves.

### 2.5. SEM Measurements

Scanning Electron Microscopy (SEM) images were obtained using Phenom Pro microscope (Phenom World, Eindhoven, The Netherlands) with backscattered detector and an operation voltage of 10 kV. Samples were not coated with a gold or carbon layer before imaging.

### 2.6. X-ray Diffraction (XRD) Study

X-ray diffraction experiment were performed with a Siemens D5000 diffractometer (Siemens AG, Munich, Germany) at RT equipped with CuK_α_ source and a rear graphite monochromator. The samples were placed on a glass substrate and fixed at the table of the goniometer. The diffraction patterns obtained were analyzed with the X’Pert Highscore Plus software (Malvern Panalytical, Worcestershire, UK) in order to identify the crystallographic phases present in the studied materials.

### 2.7. Atomic Force Microscopy (AFM) and Magnetic Force Microscopy (MFM) Measurements

Atomic Force Microscopy (AFM) images were obtained with Dimension Icon atomic force microscope (Bruker, Santa Barbara, CA) working in the air in the Tapping mode. Magnetic Force Microscopy (MFM) images were acquired using the same microscope working in the lift mode. In all MFM measurements standard silicon cantilevers with Co/Cr coating with a nominal spring constant of 2 N/m were used. Before scanning the cantilevers, they were magnetized with a small magnet. All MFM phase images were taken with the lift (surface‒tip distance) of 250 nm.

## 3. Results

The metal derivatives of butadiene rubber-based PhCP were chosen as the model systems of used CFP. PhCP material presented in this paper was characterized before in our previous works [33,34,35] using solid state NMR, FTIR spectroscopy, elemental analysis, cation-exchange capacity measurement, potentiometric titration, and SEM analysis. The general characteristics of PhCP is presented in Table 1.

**Scheme 1:**
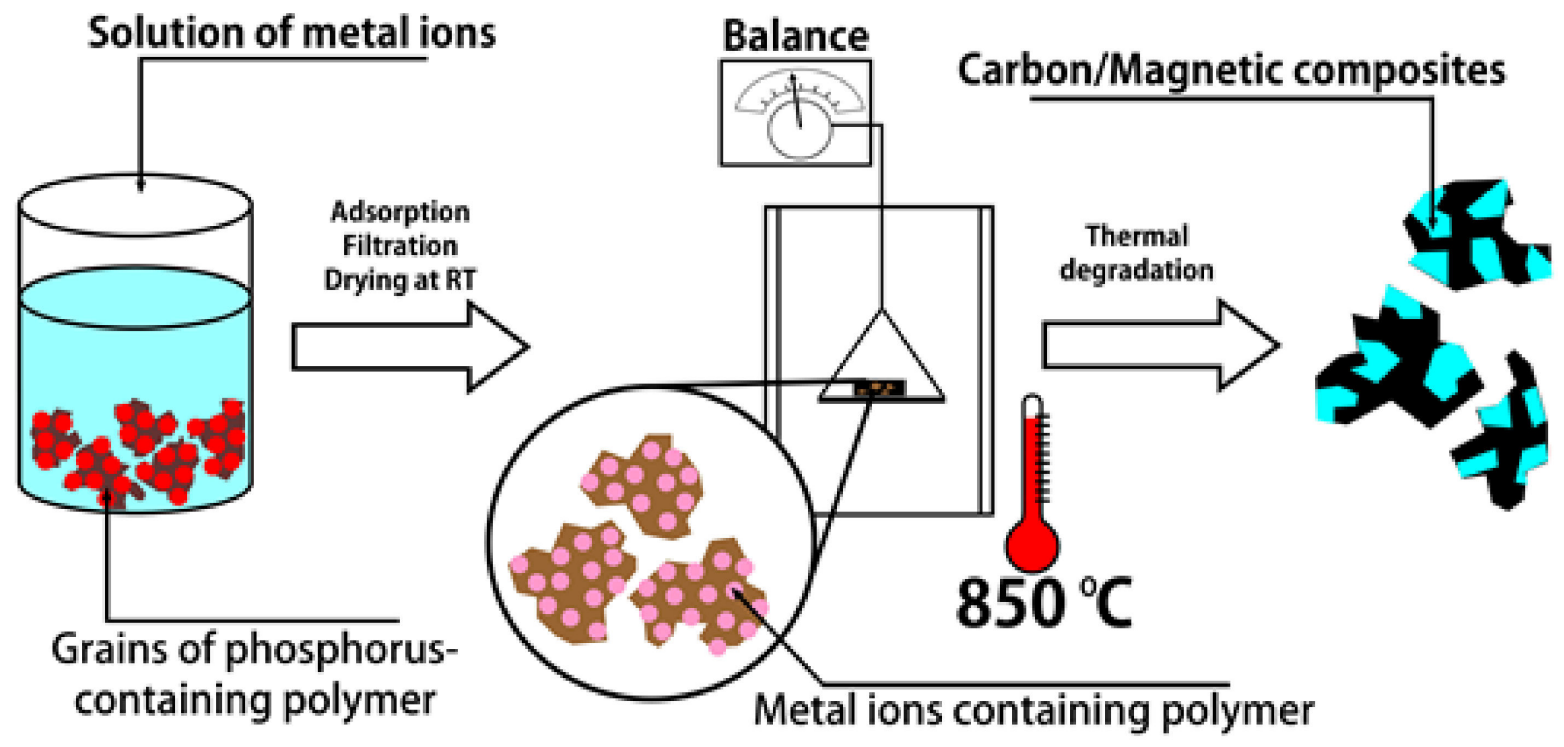
Synthesis procedure of carbon/magnetic composites.

**Figure 1:**
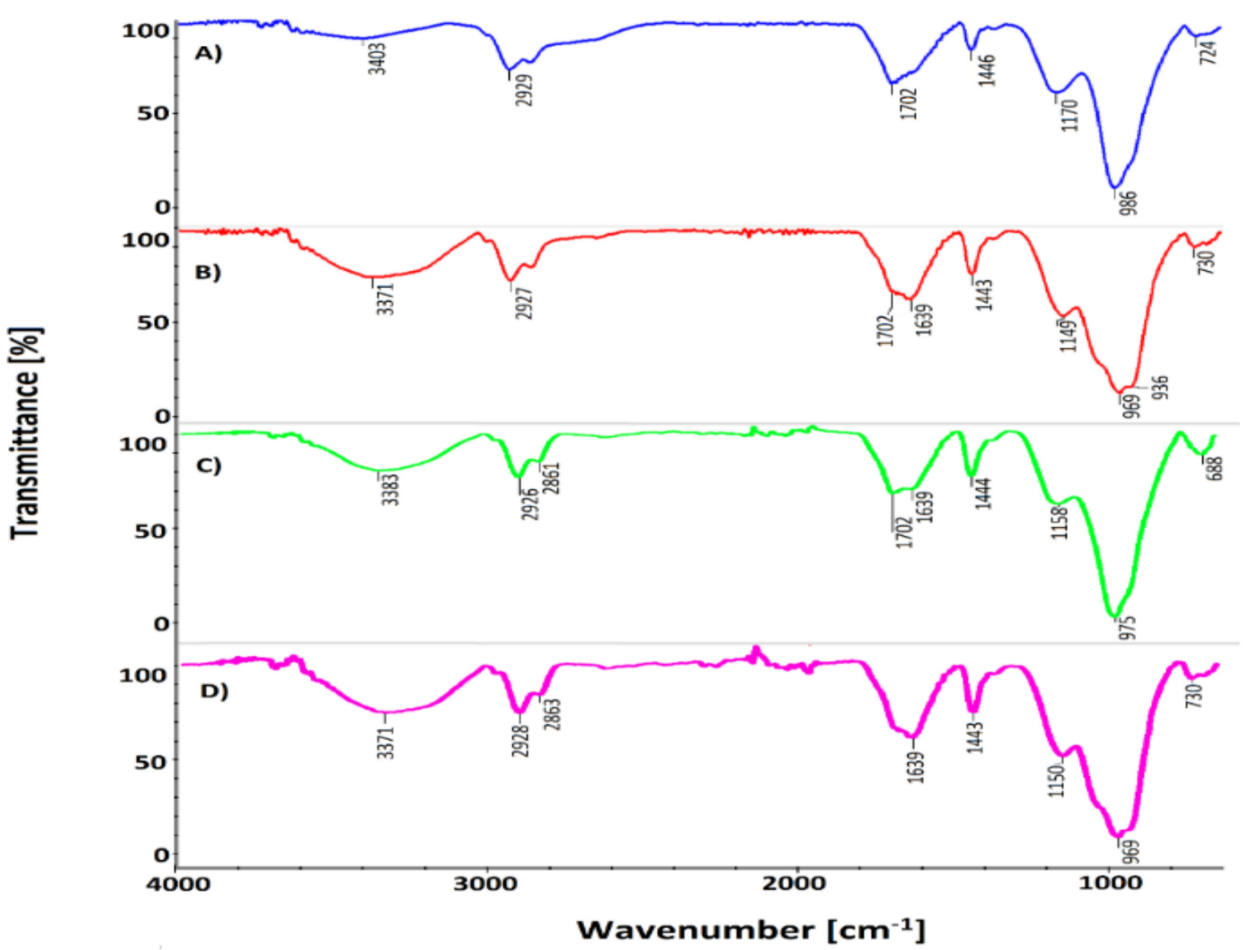
Normalized transmittance FTIR spectra of: **A**) PhCP, **B**) Ni-PhCP, **C**) Fe-PhCP, and **D**) Co-PhCP.

**Figure 2:**
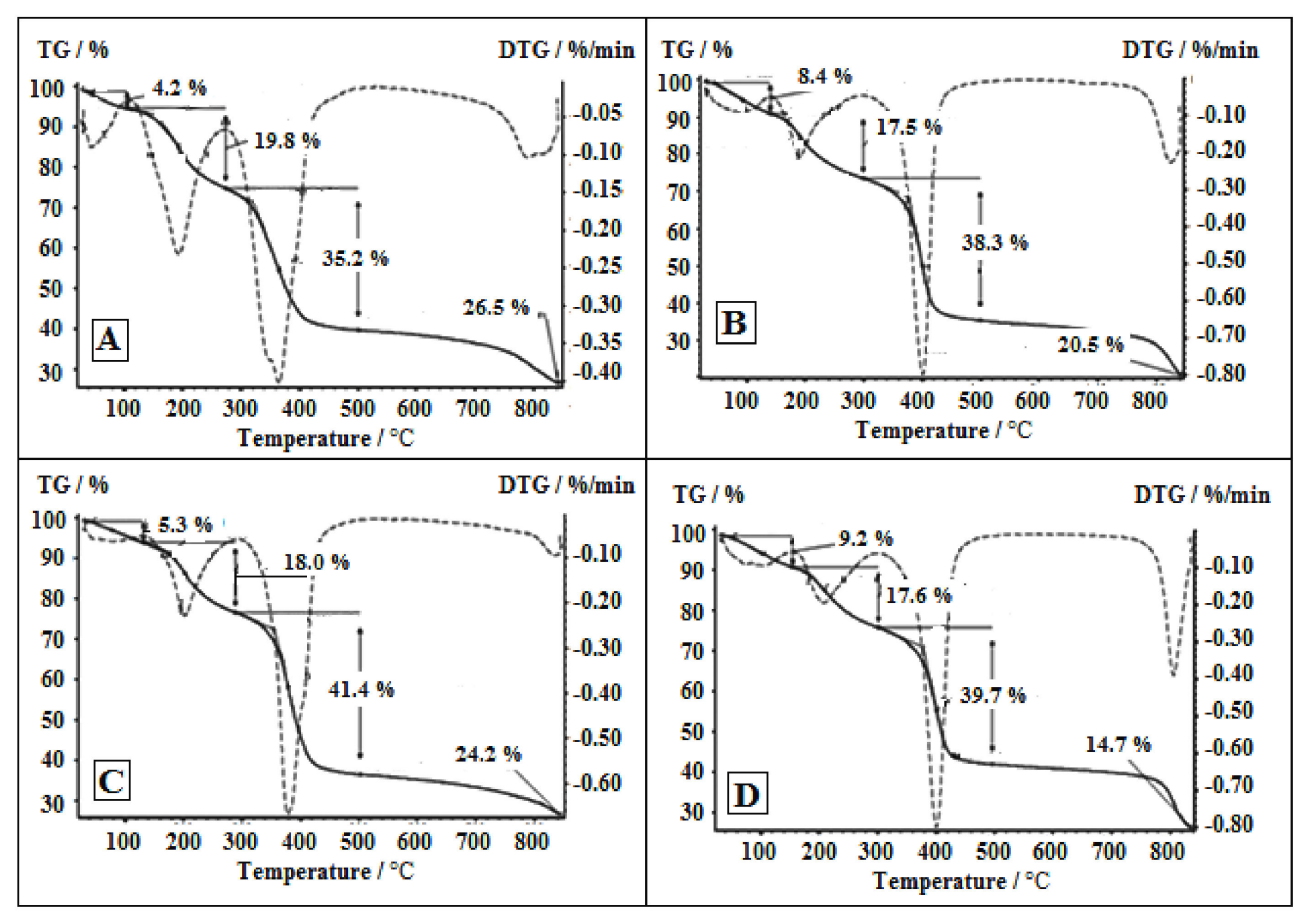
TG/DTG curves of the PhCP (**A**), Co-PhCP (**B**), Fe-PhCP (**C**), and Ni-PhCP (**D**). Heating rate: 1 K·min^−1^.

The cross-linked structure of PhCP implies its insolubility in both aqueous solutions and organic solvents. The porous structure and the presence of the main functional groups promotes the efficient sorption of metal ions (Co^2+^; Ni^2+^; Fe^3+^). We propose the usage of an efficient one-step thermal degradation process to transform used CFP into carbon/magnetic composites (CMC), which is schematically shown in Scheme 1. The amounts of the adsorbed metal additives in the PhCP were determined to be equal to 122 mg/g for Co^2+^, 130 mg/g for Ni^2+^ and 95 mg/g for Fe^3+^. Involvement of functional groups of PhCP in the adsorption of metal ions was confirmed by FTIR spectroscopy (Figure 1). The metal derivatives of PhCP were subjected to a standard thermal degradation process (TD) (Figure 2), and as a result, CMC containing a given metal obtained: Co-CMC; Fe-CMC, and Ni-CMC. Structure and magnetic properties of the obtained CMC were investigated by FTIR (Figure 4), SEM (Figure 5), XRD analysis (Figure 6), and AFM-MFM techniques (Figure 7). Similar experiments were carried out for non-magnetic CM, which were obtained by thermal degradation of native PhCP. CM were investigated by FTIR (Figure 4) and SEM analysis (Figure 5).

### 3.1. FTIR Spectra of PhCP and Its Metal Derivatives

The FTIR spectra of the PhCP and all metal derivatives are shown in Figure 1. In the spectrum of native PhCP (Figure 1A) the IR bands in the region of 1200–1150 cm^−1^ can be assigned to –P=O group vibrations, while the bands slightly below 3000 cm^−1^ (C–H stretching vibration) and at about 1446 cm^−1^ (C–H bending vibrations) to C–H vibrations of polymer main chains. The band at about 3394 cm^−1^ is attributed to OH vibration in –PO(OH)_2_ groups. IR band at 986 cm^−1^, corresponds to C–O–P bond and indicates attachment of –PO(OH)_2_ groups to the polymer chains [20,33]. Mutual comparison of the bare PhCP and its metal forms (see Figure 1B–D) revealed some differences in the region between 800–1200 cm^−1^. IR bands in the range of 1000–800 cm^−1^ are slightly shifted towards shorter wavenumbers indicating formation of ‒P(O)(OH)O^−^ anions. Furthermore, absorption of P=O groups seems to be more pronounced (1158 cm^−1^). All these changes indicate ion-coordination interaction of metal ions with the polymer functional groups, which is characteristic for the other polymers with similar functional groups ([36,37]).

### 3.2. TD of the PhCP and Its Metal Forms (Co-PhCP, Fe-PhCP and Ni-PhCP)

TG/DTG curves of the PhCP and all metal derivatives (Co-PhCP, Fe-PhCP, and Ni-PhCP), indicating the degradation stages and weight losses were are shown in Figure 2. The degradation of the samples proceeds in three stages, irrespective to its ion forms (H^+^ or Me^n+^). Moreover the degradation temperatures characteristic for the studied derivatives are different that is common for phosphorus-containing polymers [36,37,38]. The characteristic temperature of the first stage is situated in the range between 36 and 105 °C and the weight loss is varied around 4.2 wt.% for the PhCP. Weight loss at this stage is caused by the elimination of physically-adsorbed water incorporated inside the pores and water hydrogen-bonded with the functional groups. The first stages for degradation of the metal derivatives of PhCP were recorded in relatively wide temperature ranges (36–141 °C for Co-PhCP; 36–131 °C for Fe-PhCP; and 36–155 °C for Ni-PhCP) and the weight losses were bigger in comparison to PhCP. Metal ions could create aqua [Me(H_2_O)_6_]^n+^ and hydroxo [Me(H_2_O)_x_(OH)_6−x_]^n+^ complexes in water solutions and they are able to be adsorbed in the same form [39]. Thus, in the metal forms of PhCP water content seems to be bigger than in the parent PhCP. Moreover, the results show that the weight loss differ among the samples: Co-PhCP (8.4%); Fe-PhCP (5.3%); and Ni-PhCP (9.2%), which is caused by different amounts of adsorbed metal ions in the polymer structures. The second decomposition stages are characterized by a very large changes in TG curve slopes and a great weight loss for all the samples. The weight losses of the metal derivatives of PhCP are lower than for the native form of PhCP. The second stage is based on dehydratation and anhydridization processes of phosphonate and phosphate groups and elimination of hydrochloride from the polymer structure. The HCl released in the decomposition process may react with metal ions to form metal chlorides and finally release them from the structure. The third weight loss occurs above 300 °C and is characterized by a very sharp change on TG curve slopes. The weight loss has increased to the level of 35–42% and can be attributed to the elimination of the side groups from the polymeric chains, random depolymerization, and detachment of large organic molecules formed in the second stage. In the third stage all processes characteristic of a high temperature degradation like cracking and gasification occur as well. The obtained results show that metals derivatives of PhCP lose their weight relatively more efficiently than native PhCP. This can be explained by the fact that in a wide temperature range the metal ions incorporated into polymeric matrix will be already present in the oxide form, which shows the catalytic effect during the degradation process. Finally, the residuals of metal derivatives of PhCP after TD were specified as CMC.

### 3.3. TD Kinetics of the PhCP and Its Metal Derivatives

According to non-isothermal kinetic theory, thermal degradation of polymers can be described by the following function:

(1) $$\frac{d\alpha }{dT}=\frac{1}{\beta }Ae^{\frac{-E_{a}}{RT}}f(\alpha )$$

where *f*(α) is a differential expression of a kinetic model function, *α* is a fractional mass loss, *β* is a heating rate, *E_a_* is an activation energy, *A* is a pre-exponential factor for a given decomposition reaction, and T is temperature, and R is the general gas constant. The fractional mass loss ranges from 0 and 1 and expresses the reaction progress as a function of time or temperature. For non-isothermal TG analysis, the fractional mass loss at any time is equal to:

(2) $$\alpha =\frac{m_{0}-m_{T}}{m_{0}-m_{\infty }}$$

where *m_0_* is the initial sample weight, *m_T_* is the sample weight at temperature *T*, and *m_∞_* is the final sample weight. In this study, Ozawa–Flynn–Wall (OFW) ([40,41,42] and Friedman [43] methods were applied to explain the thermal degradation, by obtaining the activation energy and pre-exponential factor for the thermal degradation of each sample. The kinetic parameters were calculated using a Netzsch Thermokinetics Software (Netzsch, Selb, Germany) [44]. The mean values of *E_a_* and *A* for the four samples are presented in Table 2.

Kinetic parameters for the first degradation stage were calculated by the two methods and the values are approximately similar for all samples. The overall *E_a_* of the first degradation stage varied between 60.0 and 75.0 kJ·mol^−1^. Kinetic parameters values of the second thermal degradation stage are higher than for the previous stage. The third stage is characterized by highest values of *E_a_* that are 20–50 kJ·mol^−1^ higher for the metals derivatives than for bare PhCP. It clearly indicates that the presence of metal compounds is caused by the difficulties with removing the decomposition products from the reaction medium strongly influences *E_a_* values.

The third parameter of the kinetic triplet is reaction order, which was calculated from the results of the Friedman method. The reaction order was calculated using derivations from Equations (1) and (3) [45].

(3) f(α)=(1−α)n

The results are listed in Table 3.

As it can be seen in Table 3, the reaction orders were higher than 2.5 for the second step demonstrating that the mechanism of the thermal degradation was complex.

**Figure 3:**
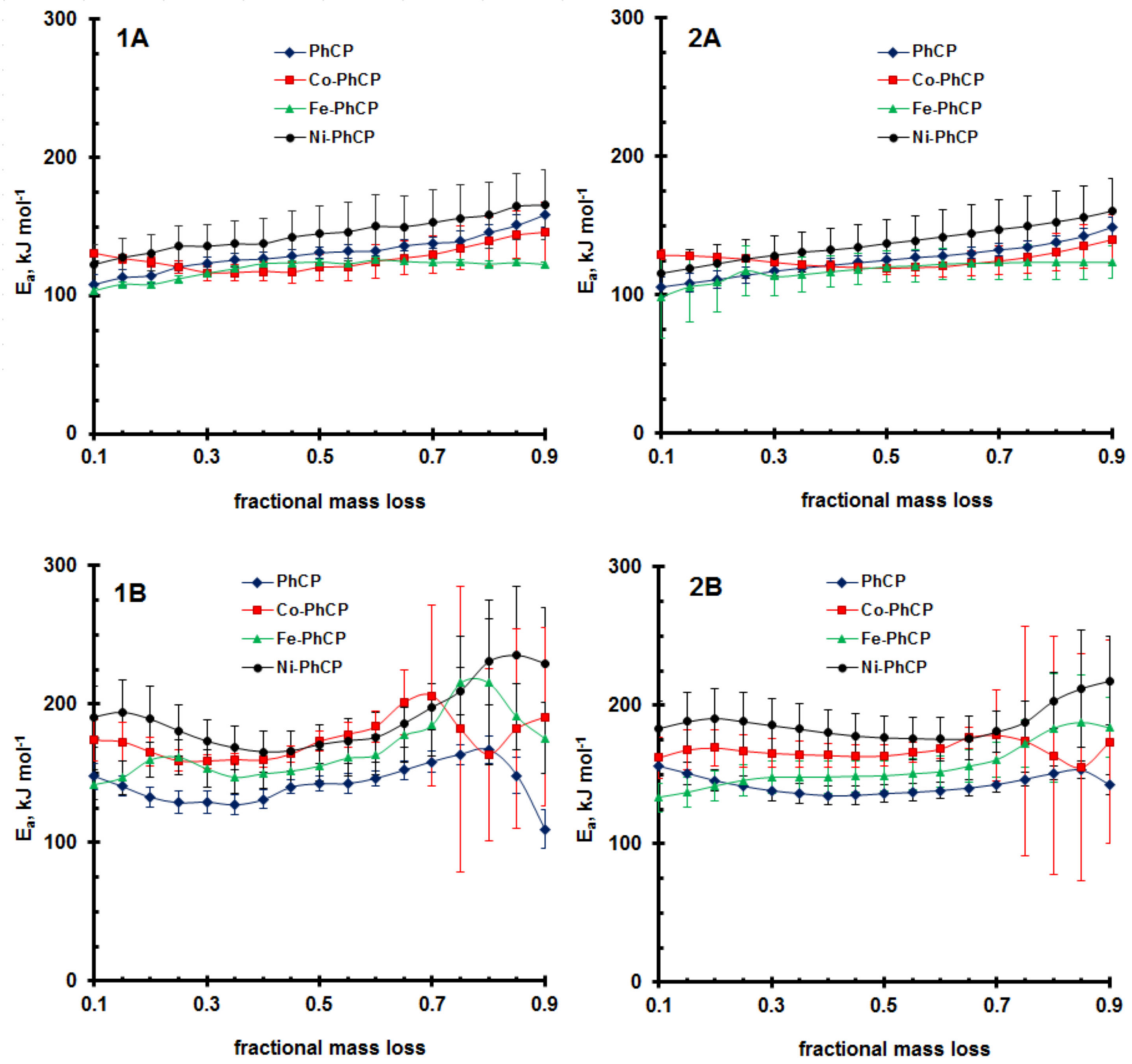
Variations of *E_a_* vs. fractional mass loss (*α*) for the second (**A**) and third (**B**) degradation stages of the samples based on Friedman (**1**) and OFW (**2**) methods.

During the thermal degradation process significant change associated with structure of the samples takes place in the second and third stages. Therefore, to have a more complete image of the thermal behavior of the studied samples, the variations of *E_a_* vs *α* (fractional mass loss) during the second and the third degradation stages were plotted in Figure 3.

The shape of the *E_a_*–α plot in Figure 3A made us suppose that during the second stage increasing the temperature causes the continuous changes of the *E_a._* These results also show that the *E_a_*–*α* plot for the metal derivatives is a little bit different than the corresponding plot for PhCP. The *E_a_* variations vs. *α* for samples, which take place in the third degradation stages are plotted in Figure 3B. The *E_a_* values change non-linearly with α parameter indicating variations in the degradation pathways. At this degradation stage, the decrease of *E_a_* (in some ranges of *α*) could also be caused by the loss of the degraded compounds formed by the detachment of the functional groups from the polymeric matrix. This variation of *E_a_*, characterized by a strong increases or decreases as a function of *α*, could be assigned either to some competitive processes or to the depolymerization in non-stationary conditions. Finally, by comparison of the *E_a_*–*α* plots obtaining two different methods, almost similar behavior in the second and the third stage was observed. That means that either the differential of Friedman or the integral method of OFW can provide a satisfactory mathematical approach to establish the kinetic parameters for the thermal degradation process.

### 3.4. Structural Characteristics of the Carbon Material and Carbon/Magnetic Composites

**Figure 4:**
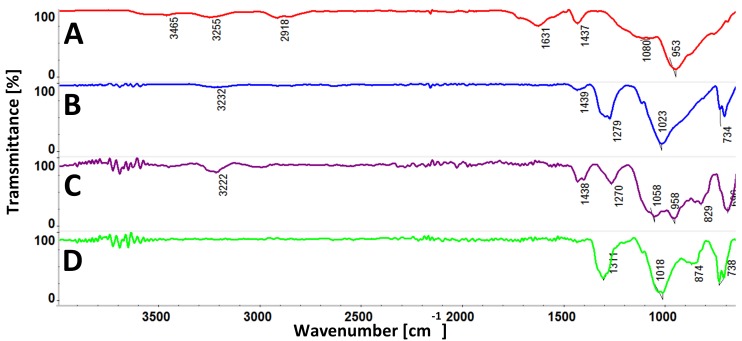
The FTIR spectra of: **A**) CM, **B**) Co-CMC, **C**) Fe-CMC, and **D**) Ni-CMC.

FT-IR spectra of CM and CMCs (Co-CMC; Fe-CMC, Ni-CMC) were shown in Figure 4. The IR spectrum of CM strongly revealed reduced absorption from organic-based compounds (Figure 4A) as the most intense bands lay below 1400 cm^−1^ and correspond to P–O, P=O, P–O–P and C–O–C groups. According to the literature, strong absorption in the mentioned region corresponds to oxidized carbons [46], carbons activated with phosphoric acid [47,48], as well as phosphorus and phosphor carbonaceous compounds. It means that PhCP after TD process is almost completely decomposed with residual phosphorus and phospho-carbonaceous compounds. In the case of CMC, practically no bands assigned to organic-based compounds/groups could be observed (Figure 4B–D). It seems that decomposition of those samples proceeded better than for CM as only IR bands assigned to inorganic compounds were detected (the bands below 1400 cm^−1^). The above mentioned bands are distinctly separated from each other due to the different strength of the bonds between metals and various phosphorus oxide anions [P_x_O_y_]^n−^ [49,50].

**Figure 5:**
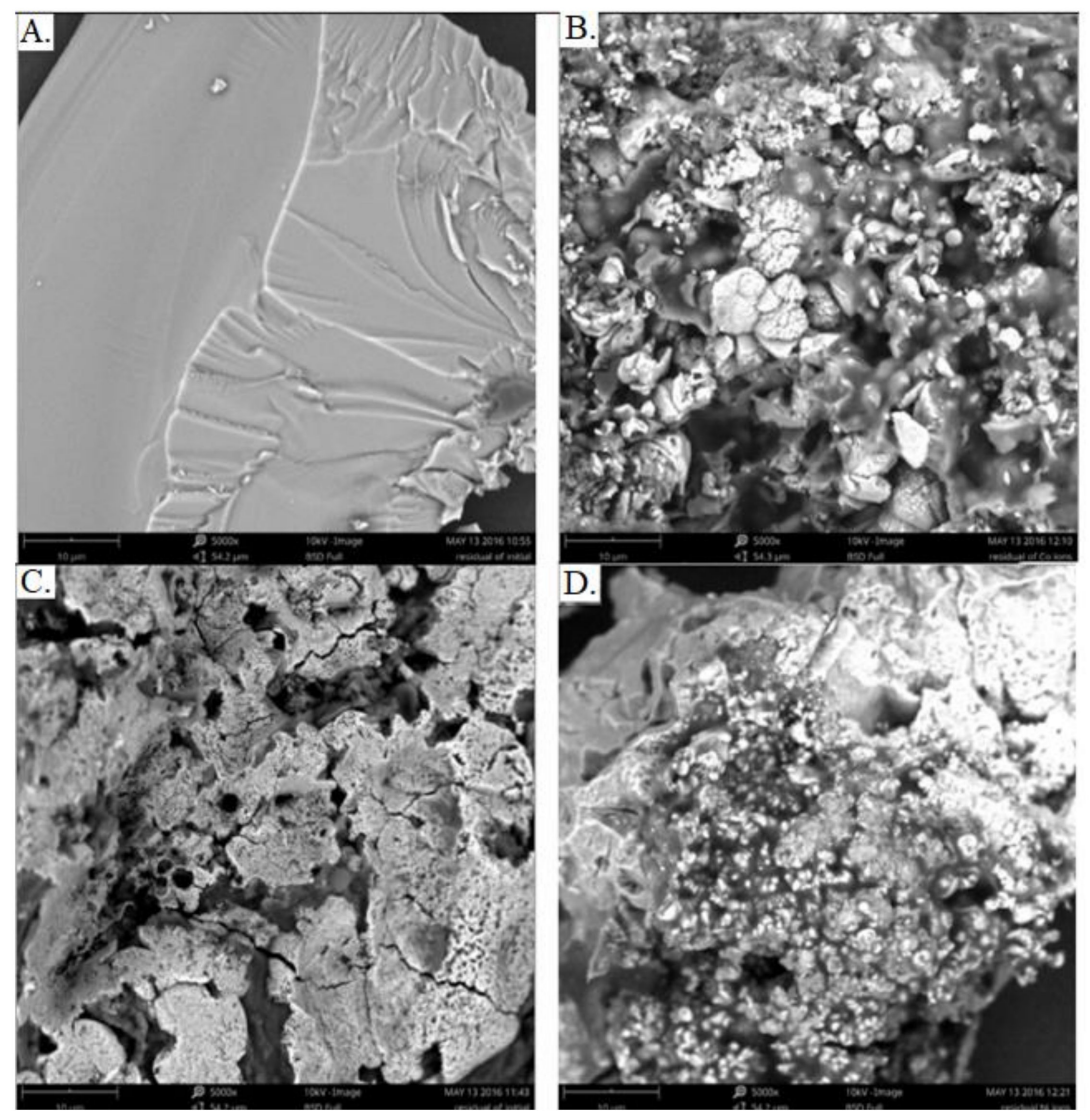
SEM images of the samples: **A**—CM; **B**—Co-CMC; **C**—Fe-CMC, and **D**—Ni-CMC.

SEM images of the samples were shown in Figure 5. The SEM images show that unlike the CM (Figure 5A.) characterized with a relatively uniform surface, all CMCs (Figure 5B–D) have bright, heterogeneous surface as a result of the incorporation of various metal compounds into their structures.

**Figure 6:**
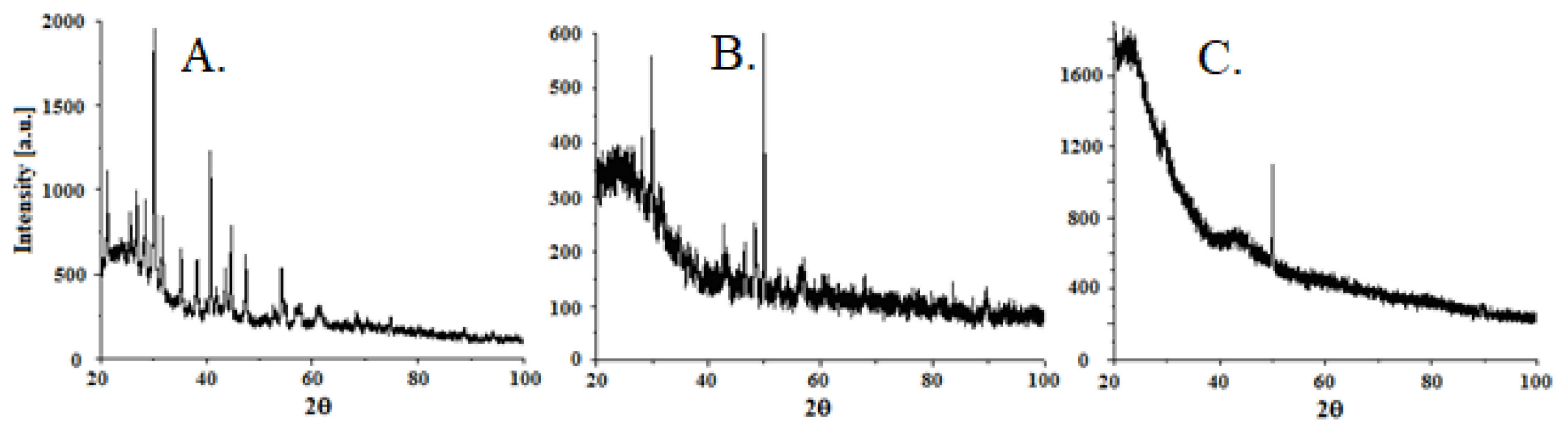
XRD patterns of the samples: **A**—Ni-CMC, **B**—Co-CMC, and **C**—Fe-CMC.

X-ray diffraction (XRD) measurements were taken for interpretation of phase and structure composition of the obtained CMC (Figure 6).

It can be seen that XRD pattern of Ni-CMC and Co-CMC contain a large number of well-resolved peaks. Analysis performed using X’Pert software revealed that the composites are of a multiphase character. The main crystalline phases present in Ni-CMC and Co-CMC were specified as: Nickel phosphate Ni(PO_3_)_2_, dinickel phosphide Ni_2_P, dinickelcyclo-tetraphosphate Ni_2_(P_4_O_12_), cobalt phosphate Co(PO_3_)_2_, and cobalt phosphide CoP, respectively. These differences between composites (Ni-CMC and Co-CMC) could be explained by the amount of metal ions in the initial functional polymers. As mentioned above, the amount of metal ions in the Ni-PhCP was greater than in the Co-PhCP. It may partially explain the fact that in the corresponding composite (Ni-CMC) three phases were identified whereas there are only two phases in the Co-CMC. Furthermore, it should be noted that dehydration and reduction reactions of phosphorus-containing functional groups (see Table 1) are responsible for the formation of the metal-phosphorus compounds. The semiquantitative analysis showed that the composition of Ni-CMC and Co-CMC are as follows: 50%-Ni(PO_3_)_2_, 12%-Ni_2_P, 39%-Ni_2_(P_4_O_12_); 76%-Co(PO_3_)_2_, and 24%-CoP. Table 4 lists the crystallographic parameters of the identified compounds in the Ni-CMC and Co-CMC. It can be noticed that the respective parameters for phosphides and phosphates of the corresponding metals are close.

In the XRD pattern of Fe-CMC broad bands in the range 39–48° and 20–35° can be noticed as well (Figure 6). The pattern also revealed strong diffraction peak at 2θ: 50° and peak at 2θ: 29° and 90°, but other peaks were not well resolved. The analysis and pattern matching can be difficult due to the quality of the diffractogram with a low signal to noise ratio that is probably related to a very low amount of iron in the composite. In general, this sample probably also contains phosphates (iron phosphates), but this requires further confirmation and additional experiments.

### 3.5. AFM-MFM Characteristics of the CM and CMCs

**Figure 7:**
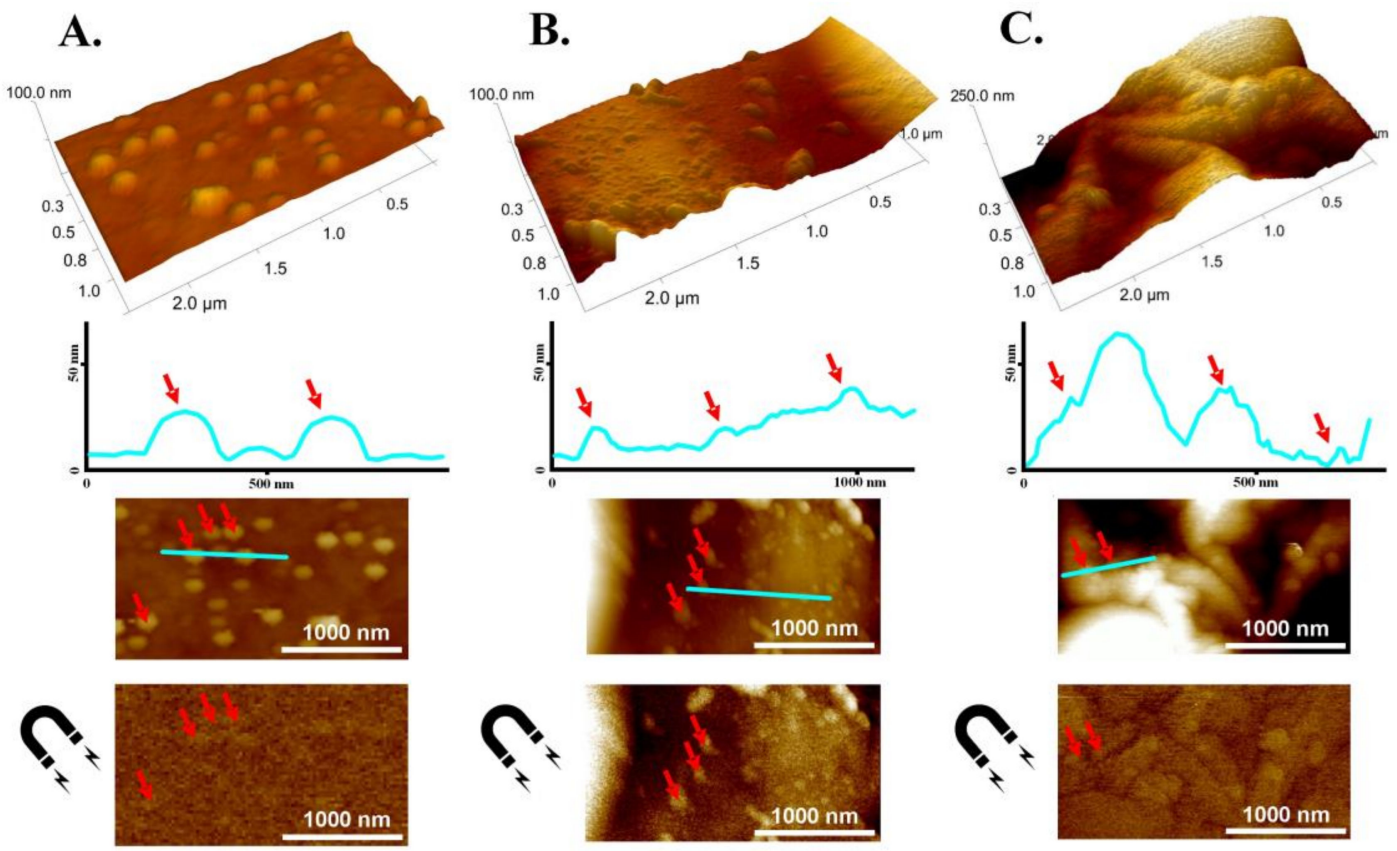
AFM topography and magnetic phase images for Fe-CMC (**A**), Co-CMC (**B**), and Ni-CMC (**C**). An arrows indicates magnetic particles.

MFM was used to confirm magnetic properties of the obtained composites. The microscope worked in the interleave mode which allowed to obtain AFM topography, phase image, and MFM magnetic phase image form exactly the same spots on the sample. All samples with carbon/magnetic composites, which are shown in AFM images reveal signal on the MFM phase image as well. This situation occurs only when samples posses a magnetic domains, which are able to interact with magnetized tip. That clearly suggest that the investigated samples exhibit relatively strong magnetic properties. AFM images taken for the Fe-CMC, Co-CMC, and Ni-CMC are shown in Figure 7. Moreover AFM technique was used to measure the average size of nanoparticles: Fe-CMC—138 ± 8 nm, Co-CMC—112 ± 14 nm, and Ni-CMC—123 ± 16 nm. 

## 4. Conclusions

Metal derivatives of butadiene rubber-based phosphorus-containing polymer (Fe-PhCP, Co-PhCP, and Ni-PhCP) were investigated as models of used crosslinked functional polymers (CFP). They were used as precursors for preparation of carbon/magnetic composites by a facile TD process. Moreover thermal behavior of Fe-PhCP, Co-PhCP, Ni-PhCP, and kinetic analysis of the TD were presented. We confirmed that degradation of the PhCP samples proceeds in three stages. The values of weight losses and kinetic parameters of the samples differ between native PhCP to all metal derivatives. A structural and morphological changes of PhCP with all metal derivatives and carbon/magnetic composites (CMC) formed after the thermal degradation were confirmed by FTIR technique. Moreover SEM analysis showed that the presence of metal-containing compounds lead to heterogenization of surfaces. XRD measurements were used to characterize the phase composition and structure of the obtained CMC. The semiquantitative analysis showed that the composition of Ni-CMC and Co-CMC reaches 50% for Ni(PO_3_)_2_, 12% for Ni_2_P, 39% for Ni_2_(P_4_O_12_); 76% for Co(PO_3_)_2_, and 24% for CoP. The differences of multiphase character of composites (Ni-CMC and Co-CMC) could be explained by the variation in the amount of metal ions in the initial functional polymers. Unfortunately, due to a very low amount of iron in the Fe-CMC samples, the XRD pattern showed only a very weak signal. AFM-MFM analyses of the samples showed that the composites have a strong magnetic properties. Thus, the offered approach is an environment-friendly and resource saving application of used CFP treatment methods. Our approach allowed us to obtain promising carbon/magnetic materials and might be useful for solving problems associated with used functional polymers.

## Figures and Tables

**Table 1:** The main characteristic of the PhCP.

Characteristic	Type
Polymer matrix structure	Cross-linked butadiene rubber
The main functional groups	Phosphonate (−OP(O)(OH)2); Phosphate (−P(O)(OH)2)
Other functional groups	>CH–Cl; >CH–OH
Ionic form	H+
Total exchange, equiv/kg of dry polymer	9.3
Ionization constant: pKa1, pKa2	4.4; 8.6
Size of particles, mm	0.30−0.40
Surface morphology	Porous and rough structure

**Table 2:** The kinetics parameters obtained by Friedman and OFW methods for PhCP and all metal forms (Co-PhCP, Fe-PhCP, and Ni-PhCP).

Samples	Step I				Step II				Step III			
	*Ea*, kJ·mol^−1^		lg*A*, s^−1^		*Ea*, kJ·mol^−1^		lg*A*, s^−1^		*Ea*, kJ·mol^−1^		lg*A*, s^−1^	
	Friedman	OFW	Friedman	OFW	Friedman	OFW	Friedman	OFW	Friedman	OFW	Friedman	OFW
**PhCP**	62.4 ± 1.6	63.5 ± 2.2	7.14 ± 0.40	6.82 ± 0.96	131.4 ± 4.3	125.4 ± 4.6	11.89 ± 2.37	10.36 ± 3.06	141.8 ± 5.1	142.9 ± 5.6	9.04 ± 1.60	9.41 ± 1.48
**Co-PhCP**	71.7 ± 8.4	75.4 ± 7.6	7.56 ± 3.09	8.06 ± 3.59	127.3 ± 12.2	126.0 ± 12.0	11.11 ± 1.63	10.72 ± 0.79	175.0 ± 9.0	167.4 ± 6.7	10.18 ± 2.81	10.00 ± 1.56
**Fe-PhCP**	66.5 ± 21.9	70.1 ± 22.5	5.91 ± 1.66	6.14 ± 1.75	119.7 ± 8.7	117.8 ± 11.5	9.47 ± 0.83	9.22 ± 0.98	167.9 ± 14.4	155.7 ± 11.62	10.89 ± 2.85	9.55 ± 2.17
**Ni-PhCP**	73.0 ± 4.0	75.6 ± 3.7	7.67 ± 2.25	7.51 ± 2.54	145.0 ± 20.5	137.7 ± 17.06	11.53 ± 1.20	11.18 ± 1.62	190.5 ± 17.0	187.4 ± 15.1	12.37 ± 2.66	12.14 ± 1.61

**Table 3:** Reaction order obtained by Friedman method for PhCP and all metal forms (Co-PhCP, Fe-PhCP, and Ni-PhCP).

Samples	Step I	Step II	Step III
**PhCP**	0.68	2.91	1.86
**Co-PhCP**	1.00	3.28	0.50
**Fe-PhCP**	1.30	2.88	1.69
**Ni-PhCP**	0.50	3.20	0.83

**Table 4:** Lists the crystallographic parameters of identified compounds in the Ni-CMC and Co-CMC.

Compound	The Lattice Parameters (nm)			Crystal System	Space Group	Space Group Number
	a	b	c			
Ni(PO3)2	1.11	0.82	0.98	Monoclinic	C2/c	15
Ni2P	0.86	0.86	0.34	Hexagonal	P-62m	189
Ni2(P4O12)	11.61	8.22	9.83	Monoclinic	C2/c	15
Co(PO3)2	1.12	0.83	0.99	Monoclinic	C2/c	15
CoP	0.51	0.33	0.56	Orthorhombic	Pnma	62
